# Effect of Wax Composition and Shear Force on Wax Aggregation Behavior in Crude Oil: A Molecular Dynamics Simulation Study

**DOI:** 10.3390/molecules27144432

**Published:** 2022-07-11

**Authors:** Shuang Wang, Qinglin Cheng, Yifan Gan, Qibin Li, Chao Liu, Wei Sun

**Affiliations:** 1 Key Laboratory of Ministry of Education for Enhancing the Oil and Gas Recovery Ratio, Northeast Petroleum University, Daqing 163318, China; wangshuang517@163.com (S.W.); sunwei19880408@163.com (W.S.); 2 CNPC Research Institute of Safety and Environment Technology, Beijing 102206, China; ganyifan@cnpc.com.cn; 3 Key Laboratory of Low-Grade Energy Utilization Technologies and Systems, Ministry of Education, School of Energy and Power Engineering, Chongqing University, Chongqing 400030, China; qibinli@cqu.edu.cn (Q.L.); liuchao@cqu.edu.cn (C.L.)

**Keywords:** crude oil, wax aggregation, molecular dynamics, shear effect, wax component

## Abstract

To explore the influence of different wax components and the shear effect exerted by the pump and pipe wall in the process of crude oil pipeline transportation on the microbehavior of wax aggregation in crude oil at low temperatures, molecular dynamics models of binary and multivariate systems of crude oil with different wax components are established in this paper. The simulation results are compared with the existing experimental results and the NIST database to verify the rationality and accuracy of the models. By using the established binary model to simulate four crude oil systems containing different wax components, it can be found that the longer the wax molecular chain, the more easily the wax molecules aggregate. The influence of temperature on the aggregation process of wax molecules with different chain lengths is also studied. The lower the temperature, the greater the difference in wax molecular aggregation degree caused by the difference in molecular chain length. Nonequilibrium molecular dynamics is used to simulate the shear process of a multivariate system of crude oil, and the micromechanisms of the shear effect on the aggregation process of wax molecules are studied. Shearing can destroy the stable structure of crude oil, resulting in the orientation and conformational transformation of wax molecules, and obtaining the region of wax molecules sensitive to temperature and shear effects, the temperatures of which are below the wax precipitation point and the shear rate of which is lower than the maximum shear rate to prevent the molecular structure from being destroyed. At the same time, the sensitivity of wax components with different chain lengths to the shear effect is studied. The research results provide theoretical guidance for ensuring the safe and economic operation of waxy crude oil production.

## 1. Introduction

According to the statistics of relevant institutions, more than 80% of the crude oil produced in China is waxy crude oil, which is a complex multicomponent mixture that includes oil, wax, asphaltene, and water. In the process of pipeline transportation, the wax in the oil will exhibit a complex aggregation behavior with changes in temperature, pressure and external forces, which often precipitates from oil with the change in phase state, resulting in wax deposition, thereby reducing the oil flow area and increasing the flow rate in the pipeline, which greatly affects the safe operation of the pipeline. Therefore, wax deposition has always been a technical problem that needs to be solved by pipeline workers [1,2,3].

In recent years, the study of wax deposition has developed from the macroscale to the microscale. From exploring the wax deposition mechanism, observing wax crystal morphology and structure to revealing the interaction between wax molecules from the microscale, scholars have a more in-depth and thorough understanding of the phenomenon of wax deposition [4,5,6,7,8]. There is no doubt that the in-depth exploration of wax deposition is inseparable from the development of research tools. From previous indoor pipeline experiments to microscopy technology and then to molecular simulation technology, a leap in research scale has been achieved. At present, using molecular simulation technology to study wax deposition in the process of crude oil pipeline transportation has become a new technology with great flexibility. It can not only simulate crude oil systems with different components and physical properties but can also simulate crude oil systems under extreme conditions, which cannot be achieved by macroexperiments [9,10,11,12,13]. Molecular dynamics is the main method of molecular simulation technology, which relies on Newtonian mechanics to simulate the motion of molecular systems. Samples are taken from a set consisting of different states of the molecular system. Then, the configuration integral of the system is calculated, and based on the results, the thermodynamic quantity and other microscopic parameters of the system are further calculated.

At present, there are many studies on the microcosmic characteristics of waxy crude oil systems by molecular dynamics. Scholars have explored the mechanism of wax molecular aggregation and the factors affecting the wax molecular aggregation process from the perspective of molecules. Gan [14] simulated the process of wax dissolution, diffusion, aggregation and deposition in a crude oil system and analyzed the effects of different operating parameters on the microcosmic dynamic behavior of wax molecules. Li [15] studied the deposition behavior of wax crystals in crude oil on a pipe wall at various temperatures and discussed the microcosmic mechanism of pour point depressants to reduce wax deposition. Yang [16] established a multiphase system composed of asphaltene, wax, pour point depressant and crude oil and studied the effects of asphaltene and pour point depressant on the wax deposition characteristics of waxy crude oil. Through the above research, it can be found that the effects of crude oil composition (asphaltene, colloid, and water) and external conditions (temperature and pressure) on the aggregation process of wax molecules were mainly studied. In the research process, the model and simulation conditions were simplified, and wax was simplified as a long-chain alkane. However, the composition of wax is very complex, the distribution of carbon atoms of its main component n-alkane is very wide, and the microcosmic behavior of wax molecules with different chain lengths will be different. In addition, the simulation process is based on the equilibrium state, but crude oil will be subjected to a series of shear effects in the process of pipeline transportation, such as pump shear and pipe flow shear, which affect the microcosmic behaviors of wax aggregation and deposition in the crude oil at low temperatures to some extent, so that the multiphase system of crude oil is in the nonequilibrium state for a long time. Therefore, it is necessary to consider the effects of wax composition and shear force on the microcosmic behavior of wax.

Therefore, the molecular dynamics models of binary and multivariate systems of crude oil with different wax components are established to study the influence of the wax molecular carbon number and shear effect on wax molecular precipitation and aggregation. The established binary models are used to simulate four crude oil systems containing different wax components, and the influence law of wax molecular chain length on the wax molecular aggregation process is obtained. Nonequilibrium molecular dynamics is used to simulate the shear process of a waxy crude oil system, and the micromechanisms of the shear effect on the aggregation process of wax molecules are studied. The viscosity of the waxy crude oil system at various temperatures and shear rates is calculated. The sensitivity of wax molecules to temperature and shear rate is analyzed, and the sensitive region of wax molecules is obtained. At the same time, the sensitivity of wax components with different chain lengths to shear is studied. The research results provide a theoretical basis for ensuring the safe and economic operation of waxy crude oil production.

## 2. Models and Simulation

### 2.1. Models

The components in the waxy crude oil system are interrelated, among which asphaltene, colloid and water have a great influence on the precipitation and aggregation of wax, which has also been proven by previous studies. To explore the influence of wax composition and shear action on the complex aggregation behavior, the crude oil model is simplified into two parts: light oil and wax without considering the asphaltene, water and other components with less content in crude oil and excluding their influence on the wax aggregation process. The carbon number distribution of light oil is approximately C_4_~C_22_. In this paper, the light oil model is represented by C_12_, which is the light oil model used in the previous literature of our research group and has also been adopted by many scholars [14,15,16]. The main components of wax molecules are long-chain alkanes above C_17_. Researchers have used C_18_, C_20_, C_28_, C_32_ and other molecules to represent the wax molecular model in the previous literature [17,18,19,20,21]. Based on Chen’s works [22,23], the proportion of each carbon number of wax is gave. The carbon number of wax is mainly distributed in C_20_–C_30_ and C_31_–C_50_. To analyze the influence of different wax compositions on wax crystallization behavior, C_24_, C_26_, C_36_ and C_38_, which account for a large proportion and have a large difference in carbon number, are selected as wax molecular models.

**Figure 1:**
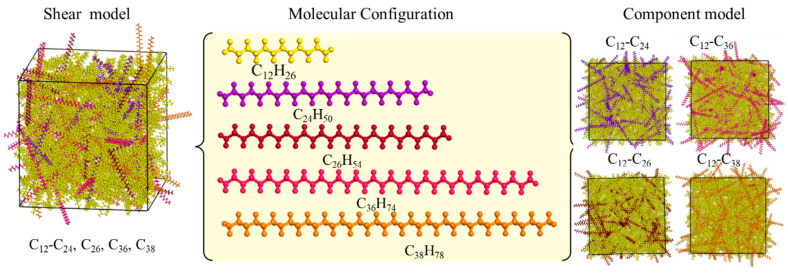
Molecular composition and model.

One is the component model, including four binary models, which are composed of four wax molecules and light oil molecules to study the influence of wax components. The other is the shear model, which is a multivariate model composed of four wax molecules and light oil molecules to explore the influence of external forces. Each model contains 750 light oil molecules and 100 wax molecules. Figure 1 shows the chemical composition of light oil and wax molecules and two types of crude oil models.

### 2.2. Force Field

**Figure 2:**
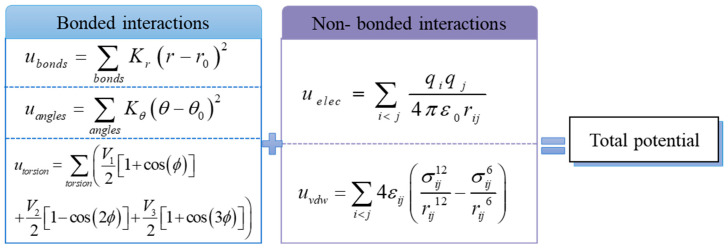
Composition of the force field.

The OPSL-AA force field is widely used to describe the microscopic characteristics of crude oil systems. It considers the interaction between each atom and can better and more accurately describe the structure and properties of a molecular system. Many studies have shown that it has strong adaptability and accuracy in describing organic molecules [15,24]. In this paper, the OPSL-AA force field is used to describe the microscopic behavior of wax in crude oil. The total potential energy of the system consists of the bond interaction potential and nonbond interaction potential. The bond interaction potential includes the bond expansion potential energy, bond angle bending potential energy and dihedral angle distortion potential energy. The nonbond interaction consists of van der Waals interaction potential energy and electrostatic interaction potential energy [25,26]. The potential function used for each force is shown in Figure 2.

Here, r, θ, and ϕ are the bond length, bond angle, and proper dihedral angle, respectively. $r_{0}$, $\theta_{0}$, and $\phi_{0}$ are reference values, $K_{r}$, $K_{\theta }$, and $V_{n}\left(n=1,2,3,4\right)$ are force field parameters. *q* is the charge of the atom and $r_{ij}$ is the intermolecular distance of atoms *i* and *j*. σ and ε are the length and energy parameters. After the force field is determined, the evolution position and velocity of each molecule in the system can be simulated. To eliminate the boundary effect caused by the scale limitation of the simulation system, periodic boundary conditions are adopted.

### 2.3. Simulation Process

**Figure 3:**
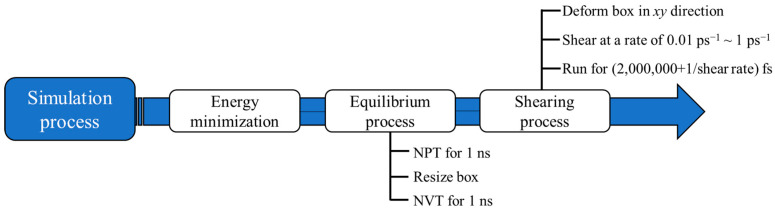
Simulation steps.

The simulation is divided into two stages: the equilibrium process and the shear process, as shown in Figure 3. Before simulation, all models need to minimize the energy to find a configuration with the minimum local potential energy.

Equilibrium process: The system is simulated between 283.15 and 353.15 K at 10 K intervals. First, the system relaxes for 1 ns under the NPT ensemble to ensure that the model reaches a stable state, and the model volume is corrected by the average density of each working condition. Then, it relaxes for 1 ns under the NVT ensemble, with a time step of 1 fs and a pressure of 0.1 MPa. A Nose–Hoover thermostat was employed to control the temperature and pressure of the systems.

Shearing process: Based on the equilibrium process, under the NVT/SLLOD ensemble, a shear rate is applied to the *xy* direction boundary of the model. The shear rate is set as 0.01 ps^−1^~1 ps^−1^, the time step is 1 fs, the cut-off radius is 12 $\overset{\circ }{A}$, and the simulation time is set as the reciprocal of the shear rate plus 2 ns. This is because the research of Liu shows that the time of reachable relaxation under the shear force field is related to the reciprocal of the shear rate [27]. In this paper, to ensure the equilibrium state of the system, the simulation time is set as the reciprocal of the shear rate plus 2 ns. The order of magnitude of the shear rate is quite different from that of the macroscopic experiment because the calculation scale of the molecular simulation is quite different from that of the macroscopic experiment, and the shear rate under this method has a certain adaptability. Through simulation, it is found that an excessively low shear rate will lead to an excessively long calculation time, while an excessively high shear rate will lead to molecular disintegration. Ali Berker et al. also confirmed this conclusion and proposed that the shear rate should be in the range of 0.001~1 ps^−1^ [28].

### 2.4. Viscosity Calculation Method

There are many calculation methods for viscosity, including the Green–Kubo formula, the Einstein equation, Muller–Platehe method, and nonequilibrium molecular dynamics [29,30,31]. At present, the method of using NEMD to calculate the viscosity of alkanes is relatively mature, and the obtained viscosity value is accurate and reliable. Berker [28] calculated the viscosity of n-hexadecane by nonequilibrium molecular dynamics and studied its rheological viscosity function. Cui [32] obtained the viscosity of decane, hexadecane and tetradecane by nonequilibrium molecular dynamics and the Green–Kubo method, respectively. Ding [33] studied the relationship between the shear viscosity and microstructure characteristics of asphalt through nonequilibrium molecular dynamics and analyzed the effects of energy, molecular composition and other factors. In this paper, nonequilibrium molecular dynamics is used to simulate the shear process of the waxy crude oil system. The SLLOD equation is used to apply Couette flow to the simulated system to obtain the viscosity. Shear boundary conditions are applied to the system, which is expected to drive the system to the steady state [34,35]. The temperature was maintained with the Nose–Hover thermostat. The SLLOD equation is expressed as [36,37]:

(1) $${\dot{r}}_{ia}=\frac{p_{\mathrm{ia}}}{m_{ia}}+\dot{\gamma }y_{i}i$$

(2) $${\dot{p}}_{ia}=F_{ia}+F_{\mathrm{ia}}^{C}-\frac{m_{ia}}{M_{i}}p_{yi}\dot{\gamma }i-\frac{m_{ia}}{M_{i}}\zeta p_{i}$$

where $m_{ia}$, $r_{ia}$, and $p_{ia}$ are the mass, position and momentum of atom a of molecule *i*. $M_{i}$, $r_{i}$, and $p_{i}$ are the mass, position and momentum of the center of mass of molecule *i*, respectively. $y_{i}$ and $p_{yi}$ are its *y* components, $F_{ia}$ represents the sum of all forces on atom a of molecule *i* due to interaction potentials, and $F_{\mathrm{ia}}^{C}$ represents all forces on atom a of molecule *i* due to any constraint forces that may be present. **i** is the *x*-unit vector.

The thermostat multiplier can be straightforwardly derived from Gauss’s principle of least constraint, which fix the peculiar kinetic energy. The thermostat multiplier *ζ* for the Gaussian isokinetic molecular thermostat is given by [38]

(3) $$\zeta =\frac{\sum_{i=1}^{N}\left(F_{i}\cdot p_{i}-\dot{\gamma }p_{xi}p_{yi}\right)/M_{i}}{\sum_{i=1}^{N}p_{i}^{2}/M_{i}}$$

The shear viscosity can be calculated from:

(4) $$\eta =-\frac{\langle P_{xy}\rangle }{\dot{\gamma }}$$

where $\langle P_{xy}\rangle$ is the *xy* component of the pressure tensor *P* and $\dot{\gamma }$ is the strain rate.

## 3. Results and Discussion

### 3.1. Model Validation

**Figure 4:**
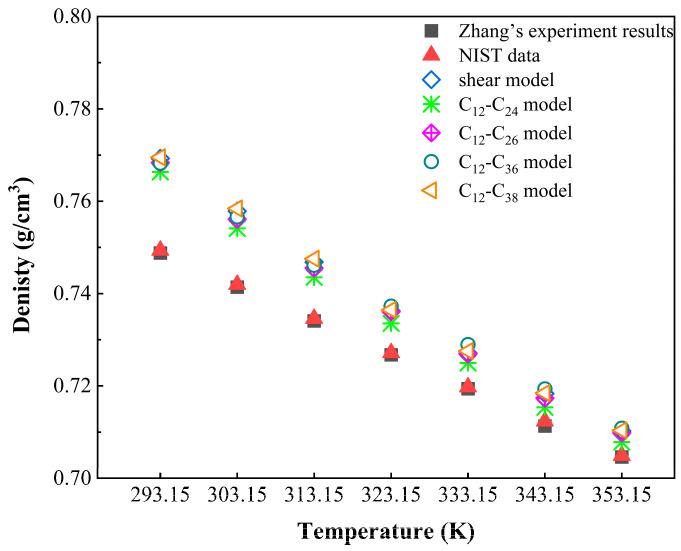
The comparison of density of C_12._

**Figure 5:**
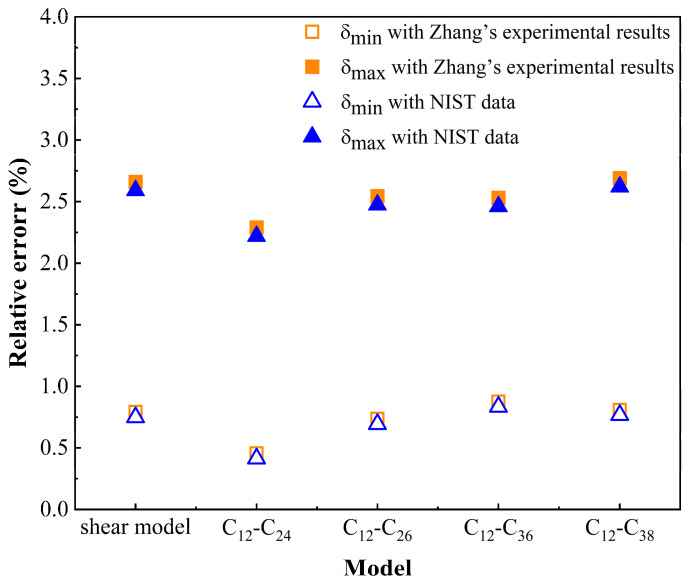
Relative error of density.

Density is an important physical parameter of crude oil and can be used to distinguish the type of crude oil. In this paper, the density of the crude oil component (C_12_) in each model is simulated in the temperature range of 293.15–353.15 K. To verify the accuracy of the molecular model, the simulated density calculation results are compared with Zhang’s experimental results and the data of the National Institute of Standards and Technology. Zhang used a U-variating tube (DMA4200) to measure the density of n-dodecane (C_12_H_26_) in the temperature range of 293 K–463 K [39]. The simulation results, Zhang’s experimental results and NIST data are shown in Figure 4. The maximum relative error between the simulated value and the experimental value is 2.69%, and the maximum error between the simulated value and the NIST value is 2.62%, as shown in Figure 5. The maximum error is within 3%. The simulation results are very close to the experimental results and NIST data, which verifies the accuracy of the model.

### 3.2. Effect of Wax Composition on the Aggregation of Wax Molecules in Crude Oil

The radial distribution function (RDF) describes the probability of other particles appearing within a certain range from a particle center, which is a parameter used to analyze the degree of aggregation of molecules in the system. The larger the value, the more aggregated the molecules.

**Figure 6:**
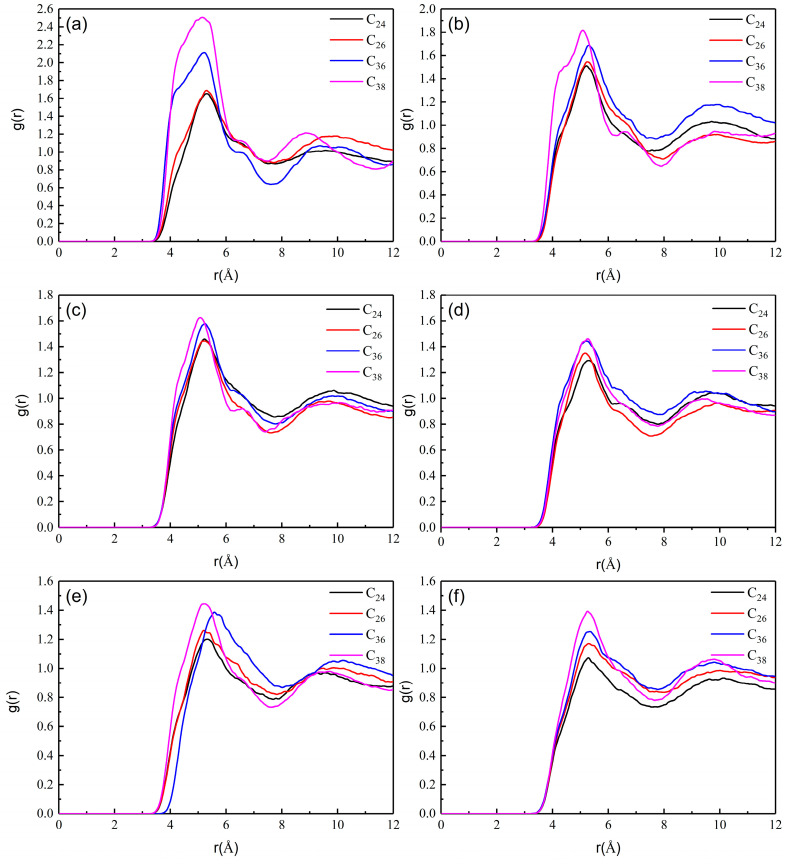
RDF of wax molecules at (**a**) 283.15, (**b**) 293.15, (**c**) 303.15, (**d**) 313.15, (**e**) 323.15 and (**f**) 333.15 K.

Figure 6 shows the radial distribution function between wax molecules in the equilibrium process of each component model at different temperatures (the aggregation degree of wax molecules is very small at 343.15 and 353.15 K, so it is not shown in Figure 6). The peak of g(r) of different wax components is approximately 5.37 $\overset{\circ }{A}$, indicating that the aggregation positions of different wax components are basically the same. The temperature and chain length have no effect on the aggregation position of wax molecules, but the peak value changes strongly due to the changes in temperature and chain length.

The peak value of RDF obviously decreases with increasing temperature. When the temperature increases from 283.15 to 333.15 K, the peak values of C_24_, C_26_, C_36_ and C_38_ all decrease, and the decrease in C_38_ is the largest, from 2.508 to 1.399. This is because the increase in temperature can intensify molecular thermal motion, which further increases the intermolecular distance, resulting in a decrease in the intermolecular van der Waals forces, thereby reducing the probability of mutual aggregation between molecules and the radial distribution function.

The peak value of RDF increases with increasing chain length because the mass of long-chain molecules is large, the van der Waals force between molecules is larger, and the aggregation between molecules is closer. The peaks of C_24_ and C_26_ are close, and the peaks of C_36_ and C_38_ are close, which also shows that the size of the peak is related to the chain length. When the temperature is low, there is a large difference between the peak values of long-chain molecules and short-chain molecules, and the difference between long-chain molecules and short-chain molecules decreases with increasing temperature. This is because the mobility of long-chain molecules is strong. As the temperature increases, the distance between molecules changes more, which makes the van der Waals force between molecules decrease more, resulting in a large change in the degree of aggregation of long-chain molecules, so the difference in the degree of aggregation caused by the molecular chain length also decreases.

### 3.3. Effect of Shear on the Aggregation of Wax Molecules in Crude Oil

#### 3.3.1. Microscopic Behavior of Wax Molecules during Shearing

**Figure 7:**
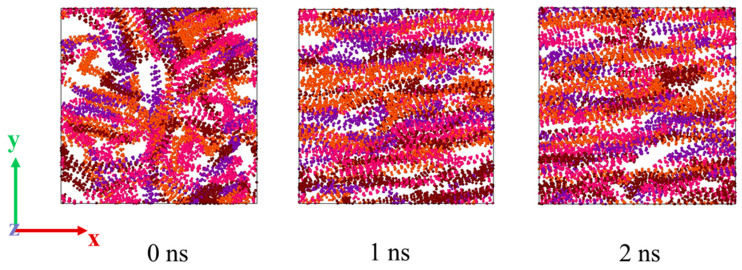
Microscopic behavior of wax molecules at a shear rate of 0.1 ps^−1^ at 293 K.

The wax precipitation point of waxy crude oil varies due to different components, and its variation range is 318.15~330.15 K. Figure 7 shows the shearing process of wax molecules in the *xy* direction when the temperature is 293.15 K and the shear rate is 0.1 ps^−1^. For the convenience of observation, only the trajectory of wax molecules is shown in Figure 7. 0 ns is the final state of the equilibrium process of the system at 293.15 K. At this time, wax molecule strands twine around each other to form a complex three-dimensional network structure. With the increase in simulation time, it can be seen that the molecular arrangement changes from a disorderly to an orderly arrangement, and the wax molecules are stretched from random coiling to a linear state, which changes the conformation of the wax molecules. At the same time, the molecular direction tends to be aligned with the direction of the shear force. The reason is that the shear force destroys the interaction between molecules and makes the molecular chain oriented and turn in the direction of the shear force field.

**Figure 8:**
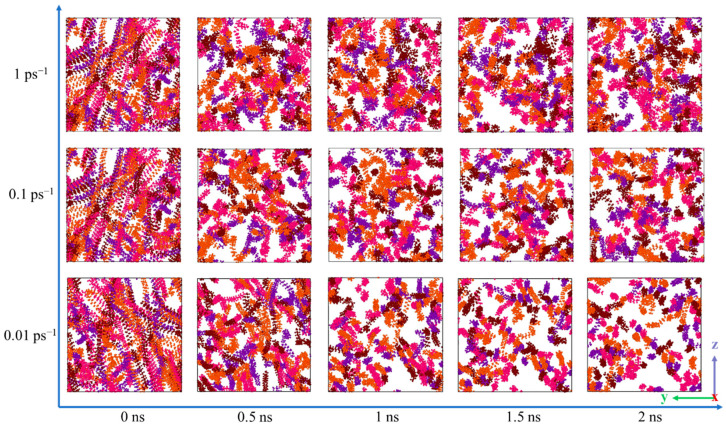
Arrangement of wax molecules in the *yz* direction at different shear rates at 293.15 K.

Although it can be seen from the *xy* direction that the molecules are arranged in a direction parallel to the shear force field, the aggregation and distribution of these wax molecules in other directions cannot be seen. Therefore, from another dimension of the simulation box, Figure 8 shows the microcosmic behavior of wax molecules in the *yz* direction at shear rates of 0.01, 0.1 and 1 ps^−1^ at 293.15 K.

As shown in Figure 8, from the horizontal point of view, with increasing shear time, the arrangement of wax molecules becomes increasingly orderly and looks more “compact”. This is because the space occupied in the *z* direction is reduced when the wax molecules are stretched from a curled state to a straight line, so the distribution of wax molecules in the *yz* direction looks orderly and compact. From the vertical point of view, with increasing shear rate, the arrangement of molecules becomes increasingly loose. This is because the large shear force destroys the interaction between molecules and destroys the formed three-dimensional network structure to disperse the precipitated wax molecules.

**Figure 9:**
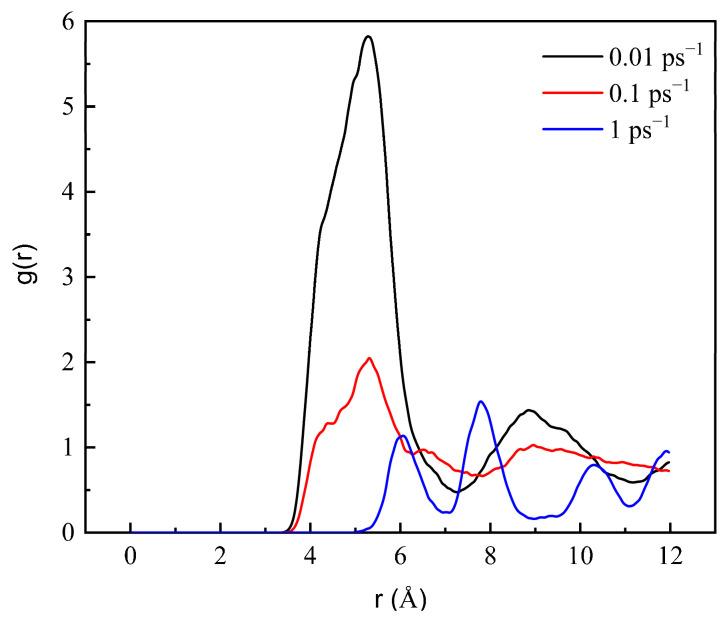
RDF of wax molecules at 293.15 K at different shear rates.

As shown in Figure 9, with increasing shear rate, the peak value of RDF obviously decreases, indicating that the aggregation degree of wax molecules decreases. It is proven that the shear force reduces the intermolecular force of wax molecules, and the aggregated wax molecules disperse continuously under the action of shear force. When the shear rate is 1 ps^−1^, multiple peaks appear, indicating that the wax crystals are decomposed by the shear force.

#### 3.3.2. Viscosity Change of the System during Shearing

Based on the RDF analysis, it is found that when the shear rate is between 0.01 and 0.1 ps^−1^, the microcosmic structure of wax molecules changes greatly. Therefore, in this range, several shear rates are selected for simulation in equal steps to explore the accurate change law.

**Figure 10:**
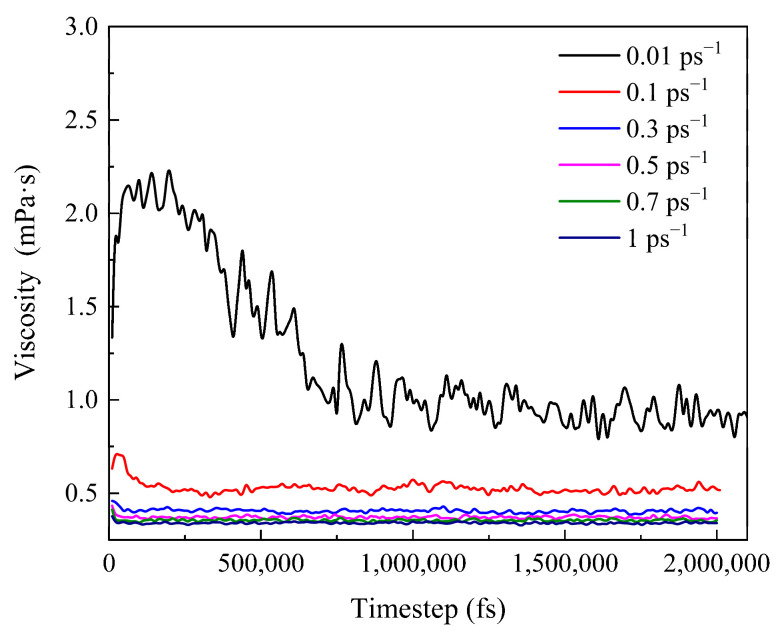
Viscosity of the system at 293.15 K.

**Figure 11:**
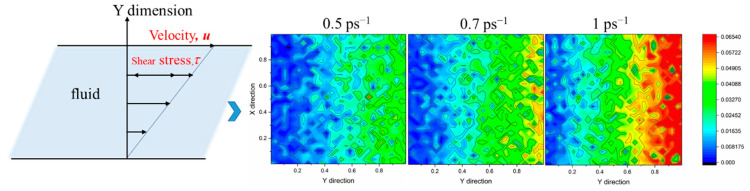
The velocity distribution of Couette flow and the velocity distribution of the system under the NEMD method.

Figure 10 shows the viscosity change of the waxy crude oil multiphase system at 293.15 K under various shear rates. The viscosity reaches stability at 1.5~2.0 ns and shows a downwards trend as a whole. This is because step 0 is the final state of the equilibrium process. With the progress of shear, the orientation and conformation of the molecules begin to change, which makes the molecular motion more intense, resulting in a decrease in van der Waals forces between the molecules, so the viscosity decreases. At the same time, it can be seen that the viscosity is different from the experimental results because of the difference in the scale and force application method used in the simulation and the experimental method. The shear force applied by the NEMD method is parallel to a plane, while the rheometer in the experiment makes the molecules produce angular rotation strain. However, through comparison, it can be found that the viscosity calculated by simulation is on the same order of magnitude as that calculated by Chen and Ali Berker in the literature, which illustrates that the NEMD method can accurately calculate the viscosity of the system and determine the relationship between viscosity and temperature or shear rate with reasonable accuracy. Figure 11 shows the velocity distribution of Couette flow and the velocity distribution of the system under the NEMD method. The response of the system to the applied shear velocity is consistent with the velocity distribution of Couette flow.

#### 3.3.3. Sensitivity of Wax Molecules to Shear at Different Temperatures

The effect of temperature on the properties of crude oil is very significant, especially the viscosity. It is necessary to explore the effect of temperature on viscosity in the shear process.

**Figure 12:**
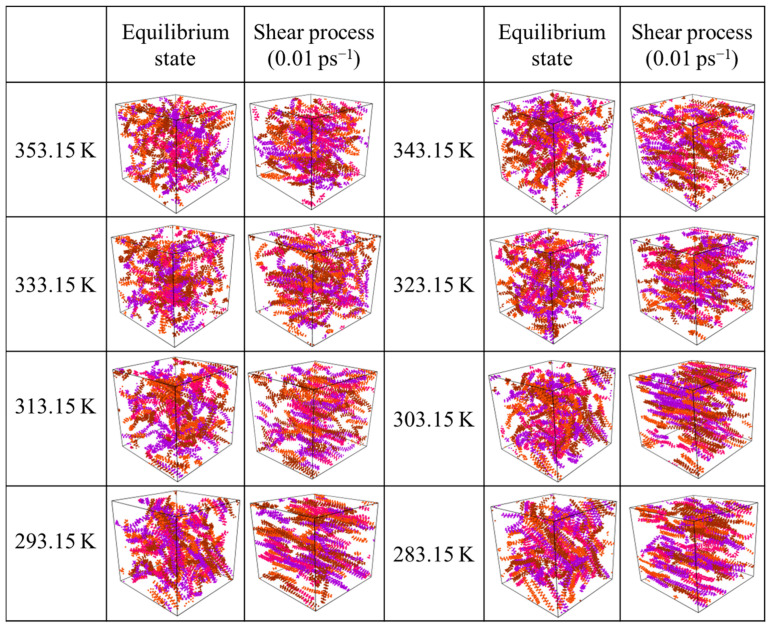
The distribution of final states of wax molecules in the equilibrium process and shear process with a shear rate of 0.01 ps^−1^ at various temperatures.

Figure 12 shows the distribution of wax molecules in the final state of the equilibrium process and shear process with a shear rate of 0.01 ps^−1^ at various temperatures. For the equilibrium process, when the temperature is high, the wax molecules are in a homogeneous and irregular distribution and exist as curly thread clusters. When the temperature drops below 323.15 K, the wax molecules gradually gather together and form an orderly arrangement in a small area. When the temperature continues to drop, wax molecular aggregates gradually change from small to large. This is because the decrease in temperature can reduce molecular thermal motion, which makes the distance between molecules closer, resulting in an increase in the intermolecular van der Waals forces; therefore, the molecules continue to gather. Under the action of the intermolecular force, the wax crystals continue to connect and gather, which makes the wax crystal particles larger, increases the degree of aggregation, and forms a three-dimensional network structure. When the shear force is applied, the wax molecules clearly stretch from random curls to straight lines, and the direction is consistent with the direction of the shear force. Especially when the temperature is below 323.15 K, the conformation and direction of the wax molecules change more obviously.

**Figure 13:**
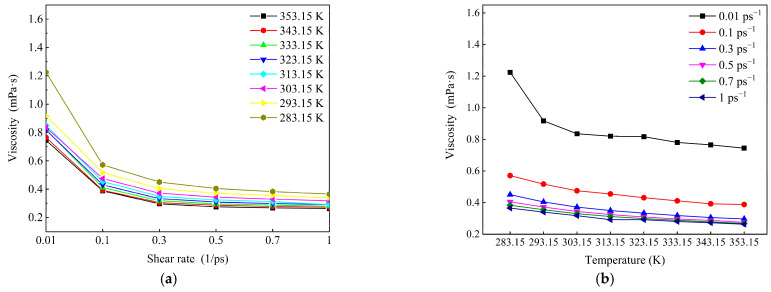
Effect of temperature on the viscosity of the system during shearing. (**a**) Variation in shear viscosity with shear rate; (**b**) variation in shear viscosity with temperature.

Figure 13 shows that the viscosity of the system decreases significantly with increasing shear rate and temperature. As shown in Figure 13a, when the shear rate increases from 0.01 ps^−1^ to 0.1 s^−1^, the slope of this section is the largest, and the decrease in viscosity is significantly greater than that of other sections. When the shear rate is greater than 0.5 ps^−1^, the decreasing trend of viscosity is gentle, indicating that the conformation of the molecules is basically linear, the direction is basically parallel to the direction of the force field, and the molecular distribution is relatively dispersed. Even if the shear rate is increased again, there is no great change. This is because the high shear rate leads to molecular decomposition, but it will not cause the mutation of viscosity. At this point, 0.5 ps^−1^ is considered to be the maximum shear rate to maintain the molecular structure that is not destroyed at this scale.

At the same time, Figure 13b shows that the viscosity decreases with increasing temperature. When the temperature is in the range of 283.15~313.15 K, the decrease in viscosity is significantly greater than that when the temperature is higher than 313.15 K. In other words, the variation in viscosity with shear rate at high temperature is less than that at low temperatures. This is because the wax crystal particles are large and the degree of aggregation is large at low temperatures, and the degree of aggregation changes greatly after being broken by the shear effect. When the temperature is high, the aggregation degree of the wax crystal itself is small, so the change range of the aggregation degree is small after being broken by the shear effect. It also shows that 323.15 K is the wax precipitation point of the system.

According to Figure 13, it can be seen that shear has a greater impact on the system when the temperature is below 323.15 K and the shear rate is less than 0.5 ps^−1^. The results are mapped to the macroscopic point of view; that is, when the temperature is lower than the wax precipitation point and the shear rate is lower than the maximum shear rate to prevent the molecular structure from being destroyed, shear has a greater impact on the system.

#### 3.3.4. Sensitivity of Different Wax Components to the Shear Effect

**Figure 14:**
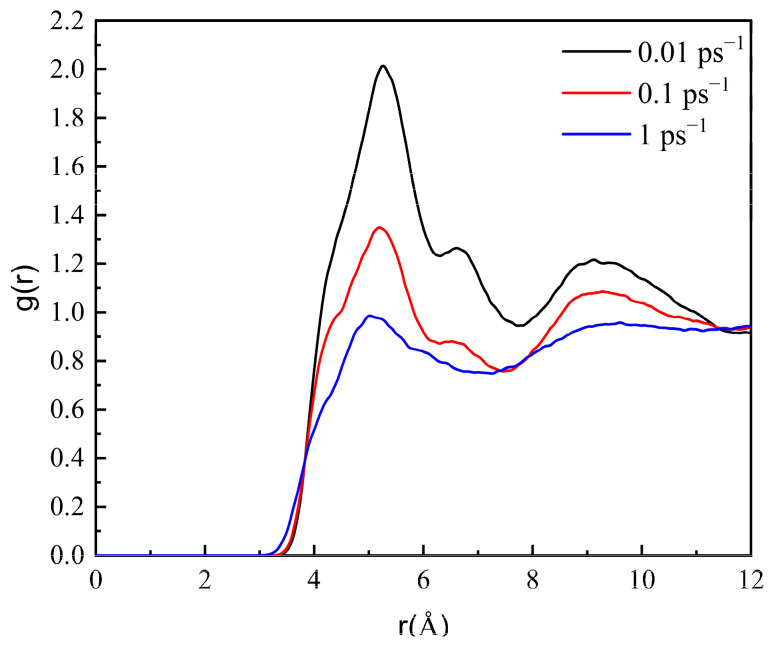
RDF of C_38_ at 283.15 K.

The aggregation of wax molecules in the binary system is simulated. Taking C_38_ as an example, the aggregation at 283.15 K is shown in Figure 14. It can be found that with the increase in shear rate, the peak value decreased significantly, which is consistent with the multivariate system.

**Figure 15:**
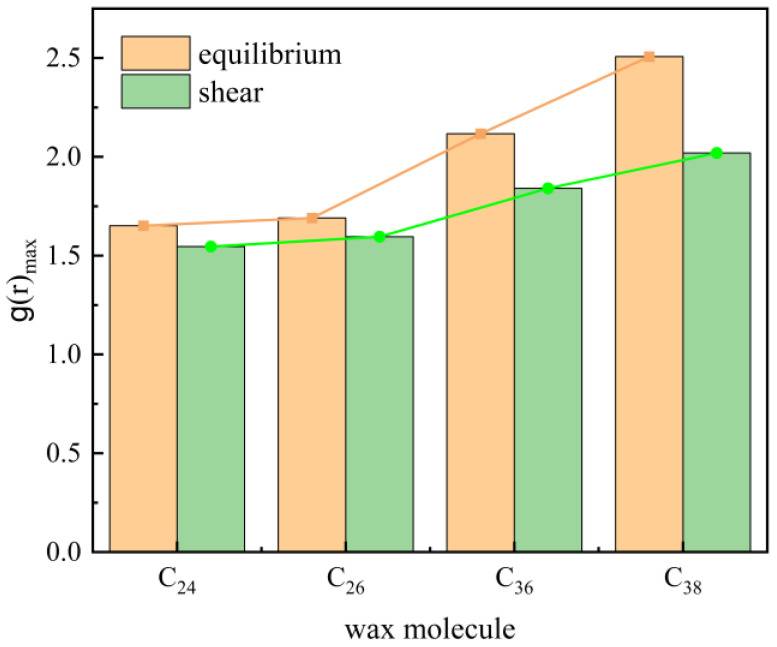
Comparison of the RDF peaks of different wax molecules before and after shearing.

According to the conclusion in Section 3.3.3, “when the temperature is below 323.15 K and the shear rate is less than 0.5 ps^−1^, shear has a greater impact on the system”. The system with a temperature of 283.15 K and shear rate of 0.01 ps^−1^ is selected for comparison, as shown in Figure 15. It can be found that with the increase in chain length, the change range of the peak value before and after shear is greater because the shear action will drive the wax molecules to expand in a straight line and make its direction parallel to the shear direction, while the conformation and orientation of long-chain molecules are more complex. To achieve this trend, the response is greater, so the change range of the aggregation state of the wax molecules is greater.

## 4. Conclusions

In this study, the effects of shear force and wax composition on wax accumulation in crude oil were studied by molecular dynamics. The main conclusions are as follows:

1. By comparing the aggregation of wax molecules with different chain lengths, it is found that the length of the molecular chain has little effect on the aggregation position of wax molecules but has a great effect on the aggregation degree. In addition, when the temperature is low, the difference in molecular chain length will lead to a large difference in the aggregation degree of wax molecules. However, with increasing temperature, the aggregation degree of all wax molecules decreases significantly, and the difference in aggregation degree caused by the molecular chain length also decreases.
2. Shearing can destroy the stable structure of crude oil, resulting in the orientation and conformational transformation of wax molecules; that is, wax molecules tend to align with the direction of the shear force, and the wax molecules are stretched from random coiling to a linear state. In a certain range, the larger the shear rate, the more parallel the direction of the wax molecules to the shear direction, and the more dispersed the distribution of the wax molecules.
3. According to the analysis of the sensitivity of wax molecules to shear action at different temperatures, it is found that the variation range of viscosity with shear rate at high temperature was smaller than that at low temperatures. This is mainly because the wax crystal particles are large and the aggregation degree is large when the temperature is low, and the aggregation degree changes greatly after breaking under the influence of shear. Furthermore, by mapping the microscopic simulation results to the macroscopic point of view, when the temperature is lower than the wax precipitation point and the shear rate is lower than the maximum shear rate to prevent the molecular structure from being destroyed, shear has a great influence on the viscosity of the system.
4. Based on the analysis of the sensitivity of different wax components to shear, it is found that wax molecules with longer chains are more sensitive to shear. The change in the aggregation degree of long-chain wax molecules before and after shearing is larger, which is due to the more complex conformation and orientation of long-chain molecules. In response to the change caused by shearing, the change in the aggregation state is larger.

In fact, the composition of crude oil and paraffin is relatively complex. Crude oil contains not only oil, but also asphaltene, colloid and other components. Paraffin has not only linear alkanes, but also a small number of alkanes with individual branches and monocyclic cycloalkanes with long side chains. In future work, it is necessary to establish a more complex and better model to obtain more accurate conclusions on the basis of considering the calculation cost.

## Data Availability

Data is contained within the article.

## Sample Availability

Samples are available from the authors.

## Nomenclature

r	Bond length
θ	Bond angle
ϕ	Proper dihedral angle
$r_{0}$	Reference bond length
$\theta_{0}$	Reference bond angle
$\phi_{0}$	Reference proper dihedral angle
$K_{r}$	Force field parameters related to bond length
$K_{\theta }$	Force field parameters related to bond angle
$V_{n}$	Force field parameters related to proper dihedral angle
*q*	The charge of the atom
$r_{ij}$	The intermolecular distance of atoms *i* and *j*
σ	The length parameters
ε	The energy parameters
$m_{ia}$	The mass of atom a of molecule *i*
$r_{ia}$	The position of atom a of molecule *i*
$p_{i\alpha }$	The momentum of atom a of molecule *i*
$M_{i}$	The mass of the center of mass of molecule *i*
$r_{i}$	The position of the center of mass of molecule *i*
$p_{i}$	The momentum of the center of mass of molecule *i*
$y_{i}$	The *y* components of molecule *i*
$p_{yi}$	The momentum of *y* components of molecule *i*
$F_{ia}$	The sum of all forces on atom a of molecule *i* due to interaction potentials
$F_{\mathrm{ia}}^{C}$	All forces on atom α of molecule *i* due to any constraint forces that may be present
**i**	The *x*-unit vector
ζ	The thermostat multiplier
$\langle P_{xy}\rangle$	The *xy* component of the pressure tensor *P*
$\dot{\gamma }$	The strain rate
